# Microarray analysis and scale-free gene networks identify candidate regulators in drought-stressed roots of loblolly pine (*P. taeda *L.)

**DOI:** 10.1186/1471-2164-12-264

**Published:** 2011-05-24

**Authors:** W Walter Lorenz, Rob Alba, Yuan-Sheng Yu, John M Bordeaux, Marta Simões, Jeffrey FD Dean

**Affiliations:** 1 Warnell School of Forestry and Natural Resources, The University of Georgia, Athens, GA 30602, USA; 2 Monsanto Company, Mailstop C1N, 800 N. Lindbergh Blvd., St. Louis, MO 63167, USA; 3 Instituto de Biologia Experimental e Tecnológica (IBET)/Instituto de Tecnologia Química e Biológica-Universidade Nova de Lisboa (ITQB-UNL), Av. República (EAN) 2784-505 Oeiras, Portugal; 4 Department of Biochemistry & Molecular Biology, The University of Georgia, Life Sciences Building, Athens, GA 30602, USA

## Abstract

**Background:**

Global transcriptional analysis of loblolly pine (*Pinus taeda *L.) is challenging due to limited molecular tools. PtGen2, a 26,496 feature cDNA microarray, was fabricated and used to assess drought-induced gene expression in loblolly pine propagule roots. Statistical analysis of differential expression and weighted gene correlation network analysis were used to identify drought-responsive genes and further characterize the molecular basis of drought tolerance in loblolly pine.

**Results:**

Microarrays were used to interrogate root cDNA populations obtained from 12 genotype × treatment combinations (four genotypes, three watering regimes). Comparison of drought-stressed roots with roots from the control treatment identified 2445 genes displaying at least a 1.5-fold expression difference (false discovery rate = 0.01). Genes commonly associated with drought response in pine and other plant species, as well as a number of abiotic and biotic stress-related genes, were up-regulated in drought-stressed roots. Only 76 genes were identified as differentially expressed in drought-recovered roots, indicating that the transcript population can return to the pre-drought state within 48 hours. Gene correlation analysis predicts a scale-free network topology and identifies eleven co-expression modules that ranged in size from 34 to 938 members. Network topological parameters identified a number of central nodes (hubs) including those with significant homology (E-values ≤ 2 × 10^-30^) to 9-cis-epoxycarotenoid dioxygenase, zeatin O-glucosyltransferase, and ABA-responsive protein. Identified hubs also include genes that have been associated previously with osmotic stress, phytohormones, enzymes that detoxify reactive oxygen species, and several genes of unknown function.

**Conclusion:**

PtGen2 was used to evaluate transcriptome responses in loblolly pine and was leveraged to identify 2445 differentially expressed genes responding to severe drought stress in roots. Many of the genes identified are known to be up-regulated in response to osmotic stress in pine and other plant species and encode proteins involved in both signal transduction and stress tolerance. Gene expression levels returned to control values within a 48-hour recovery period in all but 76 transcripts. Correlation network analysis indicates a scale-free network topology for the pine root transcriptome and identifies central nodes that may serve as drivers of drought-responsive transcriptome dynamics in the roots of loblolly pine.

## Background

Plant responses to water deficit are complex and employ a number of molecular players that act in concert to protect cells and macromolecules as the availability of water drops below critical levels. In *Arabidopsis*, rice, and other species many drought stress-inducible genes have been identified and classified into two major groups [1-3]. The first group encodes proteins thought to mediate stress tolerance, and includes late embryogenesis abundant proteins (LEAs) and chaperones, water channels, transporters and biosynthetic enzymes for compatible solutes (osmolytes), and enzymes that detoxify reactive oxygen species (ROS). The second group encodes regulatory proteins related to signal transduction, such as abscisic acid (ABA) biosynthetic enzymes, phospholipid metabolic proteins, and transcription factors (TFs) including ABA response elements (ABREs), dehydration response element binding factors (DREBs), ethylene response factors (ERFs), as well as kinases and phosphatases. Despite this knowledge base, the exact transcriptional mechanisms through which plants respond to water deficit are not completely understood and can vary significantly with respect to the species, the severity of the applied water stress, and the tissue being studied [3,4].

Phenotypic responses to water stress are most easily studied in aerial tissues and thus much of the research in this area has focused on stem and leaf tissues. Roots, however, act as the primary sensory tissue for soil water availability [5,6] and upon desiccation roots transmit information to aerial plant tissues via chemical signaling, primarily mediated by ABA [7]. Many aspects of root-to-shoot signaling remain controversial, but it is generally agreed that ABA is synthesized in the roots and released into the xylem for transport to the shoots, where it induces physiological changes, such as stomatal closure, reduced transpiration, reduced growth, and altered ethylene biosynthesis [5,7-9]. Other factors, such as cytokinins and small peptides, also originate from roots and may influence shoot responses to drying soils [6,10]. In the roots themselves, ABA has been implicated in promoting drought-induced rhizogenesis, altering the developmental program for lateral roots and root hydraulic conductivity [11-13].

Transcriptional profiling studies of above-ground tissues responding to drought stress are far more prevalent than similar analyses of root tissues, with most performed on angiosperms [14]. However, microarrays have been utilized to profile gene expression in water-stressed roots from *Arabidopsis *[15] rice [16], wheat [17], corn [18], bean [19], sorghum [20], and alfalfa [21]. These studies have identified genes that generally correspond well with those identified in aboveground tissues, including differential expression of genes involved in ABA metabolism, signaling, stress tolerance (LEAs, dehydrins, and chaperones), membrane transport (aquaporins), transporters and biosynthetic enzymes for compatible solutes (osmolytes), and enzymes for detoxifying ROS.

Species of the genus *Populus *are the most well studied in terms of providing differential expression data on responses to water deficit in trees. Recent studies have examined relationships of the transcriptome drought response as it relates to species differences [22], growth regulation [23,24] and genotype [25] with one study evaluating both roots and leaves undergoing gradual water deficit and recovery [26]. Taken together, these studies demonstrate contrasting transcriptional responses to drought with respect to species, genotypic, and diel effects. Studies using *Populus *also suggest that some drought tolerance mechanisms in roots have been conserved during the evolution of arboreal and herbaceous angiosperms, for example, the down-regulation of plasma membrane intrinsic protein (PIP) aquaporins [26]. Despite the fact that conifers are an important silvicultural species, the effect of drought stress on transcriptome dynamics in conifer roots is still unclear. However, transcriptional responses in water-stressed *Pinus *species are being investigated using a number of different genomic approaches [27-33].

The purpose of this work was to compare global transcript profiles in well-watered (WW), drought-stressed (DS), and drought-recovered (DR) roots from loblolly pine (*P. taeda*). Towards this objective we developed a collection of public genomics tools, including a set of ca. 172,000 Sanger ESTs from mostly root tissues, an annotated 26496-feature cDNA microarray for pine (PtGen2), and optimized protocols for microarray analysis of pine tissues [32,34-36]. After validating the PtGen2 microarray, we identified 2445 genes that are differentially expressed in drought-stress roots from *P. taeda *and demonstrated that the transcript population in *P. taeda *roots returns to the pre-drought state within 48 hours of cessation of the drought treatment. Weighted Gene Correlation Network Analysis (WGCNA) [37,38] identified key molecular players in the root drought response, many of which indicate similarities between mechanisms of drought tolerance in the roots of gymnosperms and angiosperms.

## Methods

### Experimental Design

Root samples were collected from 10-month old ramets, (i.e., rooted cuttings) or propagules having root structures essentially indistinguishable from those of pine seedlings. Propagules representing four genotypes (CCLONES 41201, 41369, 44686, and 45226) obtained from full-sib crosses of elite commercial germplasm were grown and harvested by the Forest Biology Research Cooperative (http://fbrc.ifas.ufl.edu) as described [32]. Established ramets were planted in plastic pots (10 cm diameter × 36 cm deep) containing 100% acid-washed fine sand, and maintained in natural daylight in a greenhouse in Gainesville, FL.

The study employed a randomized complete block, split-plot design, with genotype as the whole-plot factor and watering treatment as the split-plot factor. The drought-stress treatment (DS) withheld water until water potential in pre-dawn needles reached -1.75 MPa and needles showed visible wilting, which occurred within seven treatment days. The drought-recovery treatment (DR) withheld water until water potential in pre-dawn needles reached -1.75 MPa, followed by watering to pot capacity on day seven and a subsequent drought recovery period of 48 hours. The control treatment (well-watered, WW) propagules were watered to pot capacity every other day during the treatment period and harvested at the same time as the DS samples. Mean water potential in pre-dawn needles on the WW plants remained at -0.3 MPa ± 0.1. Tissue samples from the DR and WW plants were harvested concomitantly.

**Figure 1:**
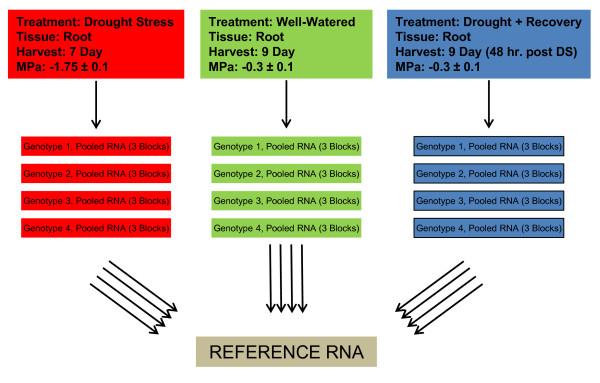
**Experimental Design**. Unrelated genotypes 1-4 represent four different clones developed from full-sib crosses of elite commercial genotypes as described in Materials and Methods. Rooted cuttings (propagules) were exposed to drought stress 10 months post planting. Mean predawn needle water potentials were determined each day and roots were harvested at indicated time points. RNA for each genotype was isolated from three individuals from different treatment blocks and pooled. Target cDNAs were synthesized and labeled with Cy3 on three separate occasions, and each was hybridized to PtGen2 twice along with Cy5 labeled reference RNA for a total of 72 hybridizations. The origin of the reference RNA is described in Materials and Methods.

Gene expression was evaluated in loblolly roots following one of three treatments (DS, DR, or WW). Four biological replicates were investigated, with each replicate comprised of RNA pooled from three individuals (Figure 1). Labeled targets from each cDNA synthesis were hybridized twice to monitor technical variation. Microarray experiments employed a reference design [39] to identify genes that exhibited treatment-specific differences in loblolly pine roots; the reference sample was comprised of total RNA from *P. taeda *roots, shoots and needles (Additional File 1).

### Quantitative PCR (qPCR)

Primer pairs were validated using a dilution series of *P. taeda *genomic DNA (80 pg - 50 ng) (Additional File 2). Validation reactions were conducted in duplicate and contained 5 uL genomic DNA, 5 uL primer pairs (0.6 uM final conc.), and 10 uL iQ™ SYBR^® ^Green Supermix (Bio-Rad Cat#170-8882). Cycling parameters and selection of primers for validation have been published previously [40]. cDNA was synthesized using Invitrogen Super Script III First Strand Synthesis System for RT-PCR (Cat No. 18080-051). Optimal cDNA concentration was determined empirically using primer pairs made for the actin ACT2 gene. The same gene was used to normalize all qPCR data. Reactions were performed in triplicate and averaged. Samples with threshold cycle (Ct) values ≥ 35 or those where the standard deviation across a triplicate was ≥ 0.5 were considered failed reactions and re-run.

### The PtGen2 Microarray

cDNA sequences for the PtGen2 microarray were derived from thirty-six cDNA libraries constructed primarily from loblolly root, stem and needle tissues [32]. A total of 25,848 (i.e., 26,496 total spots minus buffer blanks and duplicate spots) cDNA clones were amplified using 0.4 uM pSLFOR2 primer (5'-AAAACGACGGCCAGTGCCAAG-3') and 0.4 uM pSLREV2 primer (5'-GCTTCCGGCTGCTATGTTGTGTGG-3'). Reactions were incubated at 95°C for 3 min, followed by 30 amplification cycles (95°C for 30 sec, 58°C for 1 min, and 72°C for 2.5 min) and then incubated at 72°C for 7 min. All reaction products were verified by agarose gel electrophoresis. cDNAs were re-suspended in spotting buffer (150 mM PO_4 _1.5M betaine, 0.001% SDS) and printed on Corning UltraGAPS™ slides (Corning Inc., Corning, NY) using a BioRobotics Microgrid II spotter (Genomic Solutions, Ann Arbor, MI). The 384 probes in print plate #10 were re-spotted as plate #69, for use in monitoring technical variation across the array. Microarrays were printed by the Vanderbilt Microarray Shared Resource facility.

Loblolly pine 3' EST sequences have been assembled previously [32,35]. Contig consensus sequence or EST singleton sequence was used as the BlastX query to databases including NCBI nr, UniProtSProt, UniProtTrembl, UniRef, TAIR, *Medicago truncutula *Database (MT3.0), and *Oryza sativa *(OSA). Returned annotations with E-values > 1 × 10^-5 ^were ignored. A master annotation file for identification of PtGen2 probes has been included (Additional File 3). Blast2GO was used to identify gene ontology (GO) terms associated with differentially expressed genes [41].

### cDNA Synthesis, Labeling, and Hybridization

Root samples were pulverized under liquid nitrogen using a SPEX model 6850 freezer mill (SPEX, Metuchen, NJ). Total RNA was extracted from sample tissues using a modified protocol of Chang et al. [42,43] and RNA samples were DNase-treated using the Ambion TURBO DNA-*free*™ Kit (Applied Biosystems Inc., Foster City, CA). RNA concentration was determined spectrophotometrically and agarose gel electrophoresis was used to ascertain RNA quality and integrity.

Synthesis of amino-allyl and amino-hexyl modified cDNA used the Invitrogen SuperScript™ Indirect cDNA Labeling System kit (Invitrogen Corp., Carlsbad, CA). Reactions contained 20 ug of total RNA, 2 uL anchored oligo(dT)_20 _primer, 1 uL random hexamer primer, and were carried out at 45°C for 12-14 hr. Modified cDNA was purified on S.N.A.P™ columns (Invitrogen Corp., Carlsbad, CA), precipitated, and re-suspended in warm 2X coupling buffer. Cy-5 and Cy-3 (GE Healthcare Bio-Sciences Corp., Piscataway, NJ) were suspended in 80 uL DMSO and coupled to the modified cDNAs, as per the manufacturer's instructions. Cy-labeled cDNAs were purified using published methods [44], after which synthesis and labeling efficiency were monitored spectrophotometrically.

Microarray processing and hybridization protocols have been described previously [36,45]. Slides were incubated in a HybChamber™ (Genomic Solutions, Ann Arbor, MI) containing 20 uL 100 mM DTT in the humidity wells. The entire apparatus was wrapped in foil and incubated at 48°C for 14-16 hr.

### Data Analysis

Microarrays were scanned using a ProScanArray™ confocal scanner (Perkin Elmer, Waltham, MA) equipped with 532 nm and 635 nm lasers. Raw fluorescence data were processed using ImaGene, ver. 7.5 (Bio-Discovery Inc., El Segundo, CA, USA). Signal means were determined without background correction [46]. Data filtration, log_2 _transformation, normalization (print-tip Lowess), and statistical analyses were completed using BRB-Array Tools [47]. Duplicated probes on the array were treated independently during normalization and statistical analyses. Spot signals were filtered as described previously [45,48] and unigenes with ≥ 50% missing data were not included in the analysis. When necessary, missing values were imputed using the KNNimpute algorithm (K value = 15) [49]. Differentially expressed genes were identified from paired comparisons of the three treatment groups (DS vs. WW, DR vs. WW, DR vs. DS) using a random variance test [47]. The false discovery rate (FDR) was set to 0.01 and a cut-off minimum of 1.5-fold was used. Differentially expressed genes identified in the DS vs. WW comparison were used to extract the normalized log_2 _mean ratio data identified from drought-recovery samples. Weighted gene co-expression network analysis was performed as described previously [37,38] using an R-script modified for this analysis and a network threshold cut off of 0.01 (Additional File 4). Cytoscape Ver. 2.6.3 [50] and NetworkAnalyzer ver. 1.0 [51] were used to visualize the global gene network and calculate topology parameters. Candidate network drivers were identified by ranking nodes based on their degree index and also by comparing to index values determined for closeness, radiality and eccentricity.

### Sequence and Microarray Data Accession

Contig and EST sequence data utilized in probe synthesis can be accessed at the Fungen website [35]. Microarray data can be accessed at the NCBI Gene Expression Omnibus (GEO) [52] under accession GPL11184.

## Results

### Array Performance Validation

**Figure 2:**
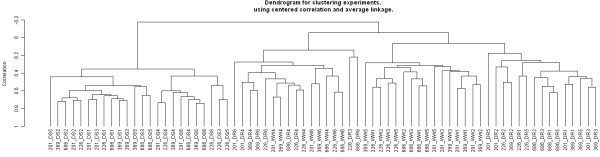
**PtGen2 Cluster Analysis Dendrogram**. Clustering was generated with BRB ArrayTools using centered correlation and average linkage. Three treatment samples are shown: WW = well watered (control), DS = drought stressed, DR = drought stressed with recovery. Each treatment identifier is preceded by the last three digits of the genotype ID. The six technical replicates are identified by the numeral following the treatment identifier, e.g. DS1, DS2.

Slide-to-slide reproducibility was verified via a hierarchical cluster analysis (centered correlation, average linkage) of all datasets from each treatment group (Figure 2) which showed a clear separation of clustering of the drought-stressed (DS) samples from well-watered (WW) and drought-recovered (DR) samples. Feature-to-feature reproducibility was verified via a correlation analysis of filtered log_2 _ratios for 384 probes that are replicated on the array. The average r-values determined for each sample (within treatments) ranged from r = 0.68 to r = 0.83, with an overall mean correlation of r = 0.78 (Additional File 5).

Quantitative PCR (qPCR) was used to validate treatment-specific expression patterns of 16 differentially expressed (DE) genes, eight of which were the most highly up- or down-regulated transcripts identified in this study, and eight of which were moderately expressed transcription factors (Additional File 6). All tested genes exhibited expression patterns that were consistent across the two platforms; however, PtGen2 underestimated the expression ratios in some cases by more than 10-fold when compared to qPCR values. Signal compression of fold-change ratios is a common phenomenon that has been demonstrated with both oligo- and cDNA microarrays [17,53] and has been attributed to the presence of cross-hybridization signals [54].

### Effect of Drought Stress on the P. taeda Root Transcriptome

**Figure 3:**
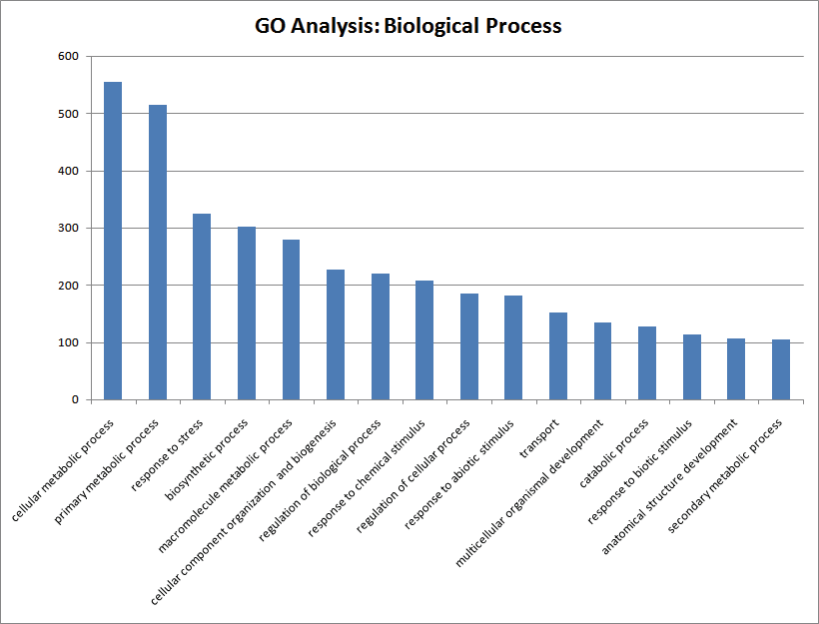
**Gene ontology (GO) analysis**. Comparison of GO terms identified from the 2445 differentially expressed genes identified in the WW and DS comparison. A) molecular function GO tags B) biological process GO tags with at least 100 entries per tag.

Comparison of drought-stressed and well-watered roots identified 2445 DE genes (Additional File 7), 1670 of which returned annotation results from BlastX and Blast2GO (E-value cut off ≤ 1 × 10^-5^). In addition to Gene Ontology (GO) terms associated with cellular and primary metabolic processes, the third most abundant GO category for the 1848 DE genes that mapped to at least one term was stress response (Figure 3). Three other relevant GO categories were associated with responses to chemical, abiotic, and biotic stresses. Table 1 lists the twenty-five drought stress-induced genes having the highest log_2 _expression ratios. Genes thought to encode a Class IV chitinase, late embryogenesis abundant proteins (LEAs), and dehydrins were among the most highly up-regulated genes; while seven genes had no apparent homology to any known proteins. Many pine genes with apparent homology to other genes associated previously with plant stress responses in angiosperms and gymnosperms were up-regulated in drought-stressed roots, including those thought to encode chitinase, dehydrin, defensin, chaperones/HSPs, water-deficit-inducible LP3, galactinol synthase (GolS), glutathione peroxidase, thaumatin, sucrose synthase (SuS), and β-glucosidase (Additional File 7). In particular, 16 DE genes thought to encode LEAs were detected and 11 were up-regulated following drought stress, as were all 15 genes identified as dehydrins. LEAs and dehydrins were also identified as hubs for the predicted global gene network (Table 2). Genes that may be involved in the production of osmolytes were also up-regulated. For example, all DE genes identified as SuS (6 probes), GolS (11 probes), or raffinose synthase (2 probes) were up-regulated after drought-stress, as were genes thought to be involved in proline biosynthesis, e.g. pyrroline-5-carboxylate synthetase (2.2.16.16).

**Table 1:** Twenty five most up-regulated genes in the DS versus WW comparison

Gene ID	**Fold **↑	Accession	Annotation	E-Value
10.2.21.6	12.1	XP_002275386.1	class IV chitinase	9.0E-60
11.2.16.23	11.8	AAT45001.1	metallothionein	2.0E-13
6.4.17.7	9.3	AAU87300.1	LEA	3.0E-18
1.3.16.12	9.2	ACA51879.1	dehydrin 2	3.0E-34
4.1.22.8	9.1	AAM28916.1	NBS/LRR	1.0E-42
6.2.23.16	8.5	AAU87300.1	LEA	1.0E-26
10.4.6.8	8.4		No Hit	
5.4.9.1	8.2	ABZ01360.1	cytochrome p450	2.0E-41
6.2.23.18	8		No Hit	
2.4.16.12	7.8	ACA51879.1	dehydrin 2	4.0E-49
3.1.16.14	7.7	AAL24147.1	cardiolipin synthase/phosphatidyltransferase	2.0E-08
8.3.7.17	6.8	Q50EK4.1	cytochrome P450 750A1	1.0E-111
12.3.21.7	6.8	AAU87300.1	LEA	4.0E-27
1.2.16.21	6.7	CAC84486.1	nodulin like-protein	1.0E-44
9.4.16.11	6.4		No Hit	
7.2.16.22	6.4		No Hit	
6.4.16.18	6.3	Q50EK4.1	cytochrome P450 750A1	1.0E-114
5.4.21.16	6.2	AAK25836.1	malate synthase	1.0E-102
8.3.2.21	6.1		No Hit	
7.2.9.10	6	ACG35207.1	dihydroflavonol-4-reductase	3.0E-31
3.4.16.12	6	Q50EK4.1	cytochrome P450 750A1	4.0E-69
9.2.15.7	6		No Hit	
3.1.11.9	5.9	AAX68990.1	LEA	6.0E-45
10.2.16.5	5.9	ABO61348.1	defensin	2.0E-35
3.2.1.7	5.9	AAG50560.1	inositol transporter 2	2.0E-60

Gene ID = unique probe address (metarow, metacolumn, row, column). Fold increases determined from normalized log_2 _mean ratios are shown for each gene along with associated BlastX accession, annotation, and E-value.

**Table 2:** Network Topology Statistics

Network Property	**Values**^**a**^
	

Nodes	1477

Edges	49997

Scale-free topology^b^	0.829

Mean shortest path length^c^	3.1

Network diameter^d^	10

Mean connectivity^e^	17.3

Centralization^f^	0.648

Heterogeneity^f^	1.91

Clustering coefficient^g^	0.811

^a^Global gene networks were generated via Weighted Gene Correlation Network Analysis (WGCNA) [37] and network properties were calculated using the *Network Analyzer *plugin [51] that was developed for Cytoscape [50].

^b ^Identifies networks that have a topology similar to other biological networks and ranges from 0 - 1 [37].

^c^Average shortest path length between two nodes.

^d^Maximum distance amongst all distances calculated between two nodes.

^e^Average number of connections made by each node.

^f^Network topology that resembles a star has a centralization of 1 and decentralized networks have a centralization of 0. Heterogeneity reflects the tendency of a network to contain hub nodes [119].

^g^The average clustering coefficient can be used to measure whether the network exhibits modular organization [120].

Up-regulated DE genes thought to encode regulatory proteins included ABA-responsive protein orthologs, aminocyclopropane (ACC) oxidases and synthase, and several transcription factors. Transcription factors (TFs) showing increased transcript abundance after drought-stress included genes with notable homology to DREBs (3.2.8.7, 4.4.14.24) and ERFs (7.3.7.6, 8.1.15.4), as well as TFs in the WRKY (7.3.15.5), Dof (2.4.15.5), bZIP (9.1.19.7), MYB (3.1.19.21), and NAC (6.2.15.1) families. Several of these are thought to be central players in signal transduction pathways involved in both drought and abiotic stress responses [1,3,55,56].

Of the 2445 DE genes, 1251 were down-regulated in DS roots. These included genes thought to encode auxin-responsive proteins, and components of cell wall and carbohydrate metabolism (Additional File 7). Numerous genes encoding putative aquaporins were down-regulated after drought stress, as were genes with homology to histones, dirigent-like proteins, cellulose synthases (CesA), expansins, pectate lyases, senescence-associated proteins, subtilisin-like and aspartic proteinases, and xyloglucan endotransglycosidases. For example, of the 18 DE genes thought to encode aquaporins (AQPs), most fell into the plasma membrane intrinsic protein (PIP) subgroup and all were down-regulated, except an *Arabidopsis *PIP2-6 ortholog (9.3.16.18). Only seventeen TFs were down-regulated and only two of those, AP2/ERF (2.2.22.4) and WRKY (1.2.18.1), have been previously associated with regulatory roles in drought response.

**Figure 4:**
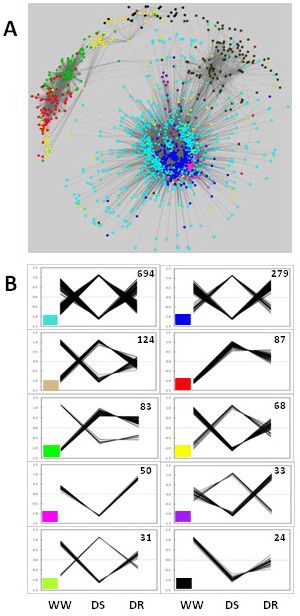
**Weighted Gene Correlation Network**. (**A**) Global gene correlation network for root tissues in *Pinus taeda*; data from well-watered (WW), drought-stressed (DS), and drought-recovered (DR) roots are shown. Ten coordinated expression modules were identified using Weighted Gene Correlation Network Analysis and the network was plotted using Cytoscape. Nodes belonging to the same co-expression module are represented via node color. Network topology statistics are shown in Table 2. (**B**) Gene expression profiles for each co-expression module are highly correlated in drought-treated pine roots. Modules are identified via the color panel at the lower left of each graph; the number of genes in each co-expression module is shown in the upper right of each graph.

**Figure 5:**
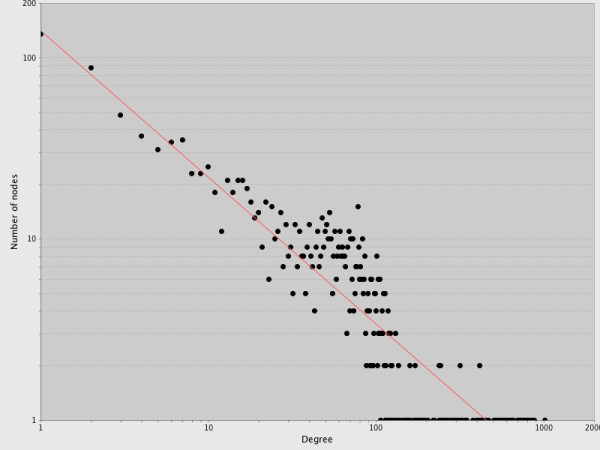
**Node degree distribution**. Number of nodes (genes) plotted as a function of their degree (the number of connections made with other nodes) shows a power-law like distribution indicative of scale-free network topology. Figure as output directly from NetworkAnalyzer [51].

Weighted Gene Correlation Network Analysis (WGCNA) [37,38] was employed to mine the 2445 DE genes for candidate regulatory genes. As would be expected for a biological system, our data predict a gene co-expression network that displays scale-free topology with inherent modular features (Figure 4A). Eleven co-expression modules (with a minimum of 30 genes each) were identified (Additional File 8). Evaluation of expression patterns from individual network modules revealed groups ranging from 24 to 694 genes that displayed coordinated changes in their transcript populations during drought stress and drought recovery (Figure 4B). As shown in Figure 5, node degree distribution for the pine gene network approximates a power-law distribution, which is a characteristic of scale-free networks [57]. Network statistics demonstrate that the pine root gene network has properties found in other biological networks (Table 2). For example, 1477 nodes were connected by 49,997 edges, representing 2.3% of the total edges possible in the network. Sparsely connected nodes in highly clustered areas are a common characteristic of the hierarchal architecture seen in many biological networks [58]. The average clustering coefficient as well as values determined for the shortest path length and smallest network diameter, are characteristic of network compactness and indicative of "small-world" networks that display high clustering and short path lengths [59].

Node centrality indices were determined and the degree topological index was used to identify putative drivers of the root co-expression network (Table 3) [60,61]. Other network centralities used to rank node significance (e.g. closeness, radiality, and eccentricity) supported the ranking determined with the degree index (data not shown). A gene of unknown function is the most highly connected node in the network while other ranked nodes are for genes that appear to encode a thioredoxin (8.3.18.12), a nucleotide binding site-leucine-rich repeat protein (NBS-LRR) (4.1.22.8), and an inositol transporter (3.2.1.7). Nodes with possible regulatory significance included those for a 9-cis-epoxycarotenoid dioxygenase (6.4.9.19), a zeatin-O-glucosyltransferase (5.4.16.5), and an ABA-responsive-like protein (7.3.13.14).

**Table 3:** Putative gene network drivers determined by Weighted Gene Correlation Network Analysis (WGCNA)

Gene ID	UniScript	**Fold **↑	Degree	Accession	Annotation	E-Value
7.4.16.13	0_7045	5.3	1023	ABK23726.1	unknown [*Picea sitchensis*]	1.0E-58
8.3.18.12	2_2955	3.3	879	EEF36951.1	thioredoxin	2.0E-57
4.1.22.8	2_8298	9.1	856	AAM28916.1	NBS/LRR	1.0E-42
3.2.1.7	0_18714	5.9	785	AAG50560.1	inositol transporter	2.0E-60
10.3.16.6	2_3115	5.8	750	AAG02215.1	class III peroxidase	8.0E-29
3.1.16.14	0_6867	7.7	745	NP_567273.1	cardiolipin synthase/phosphatidyltransferase	2.0E-08
9.4.21.14	2_5057	4.1	723	AAG23841.1	metallothionein-like protein	5.0E-11
1.2.16.21	0_6914	6.7	700	CAC84486.1	nodulin-like protein	1.0E-44
3.1.11.9	0_8943	5.9	627	AAX68990.1	LEA protein	6.0E-45
4.3.16.13	2_1036	4.2	593	ABF39004.1	phenylcoumaran benzylic ether reductase	1.0E-126
2.4.16.12	2_5467	7.8	534	ACA51879.1	dehydrin 2	4.0E-49
6.4.9.19	0_11252	2	533	BAF31905.1	9-cis-epoxycarotenoid dioxygenase	6.0E-31
11.2.16.23	2_10347	11.8	527	AAT45001.1	metallothionein	2.0E-13
3.3.21.2	2_623	5.4	526	NP_001045400.1	Probable calcium-binding protein	9.0E-31
5.4.16.5	2_8560	3.7	524	Q9ZSK5.1	zeatin O-glucosyltransferase	2.0E-30
1.3.16.12	2_5201	9.2	421	ACA51879.1	dehydrin 2	3.0E-34
11.3.5.23	0_15179	5.5	417	ACB56927.1	glycosyltransferase	4.0E-43
7.3.13.14	2_1964	3.6	417	NP_196824.1	ABA-responsive protein-like	1.0E-70
12.1.17.19	2_3445	4.4	413	ABK22613.1	unknown [Picea sitchensis]	6.0E-23
7.2.16.7	2_4960	2.7	404	ABA18653.1	glutamate decarboxylase	4.0E-84
9.4.21.15	2_131	2.7	380	XP_002530754.1	prephenate dehydrogenase family protein	0.0E+00
3.4.16.24	2_194	4.7	343	AAM28916.1	NBS/LRR	1.0E-45
6.2.16.24	0_6964	2.6	325	ACQ42253.1	SnRK2 calcium sensor	8.0E-32
2.1.8.5	0_11825	4.3	309	ABR18051.1	unknown [*Picea sitchensis*]	0.0E+00
8.4.3.18	0_17040	4.7	297	gb ABF39004.1	phenylcoumaran benzylic ether reductase	0.0E + 00

### Transcriptome Responses in Drought-Recovered Roots

**Figure 6:**
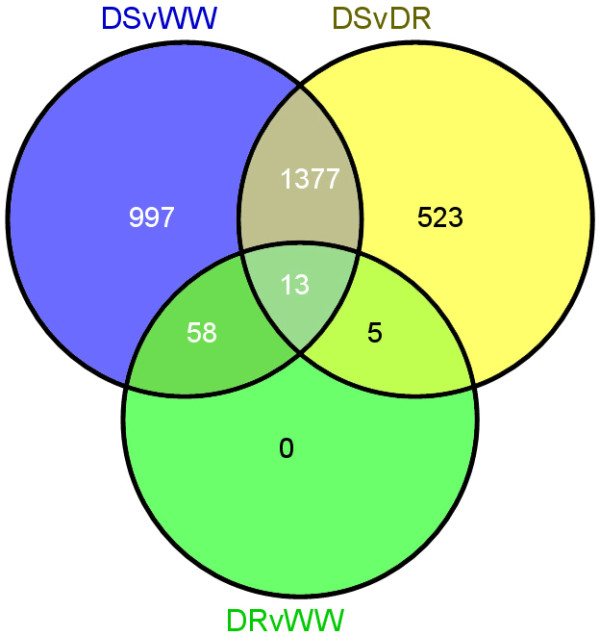
**Venn analysis of differentially expressed genes in WW, DS and DR**. Venn diagram showing overlap of differentially expressed genes identified in the three treatment comparisons. WW = well watered (control), DS = drought stressed, DR = drought stressed with recovery.

Comparison of DR roots with WW roots identified only 76 DE genes (Additional File 7), whereas comparison of DS roots with DR roots identified 1918 DE genes (Additional File 7). More than 70% of the genes in this latter set were also identified in the comparison of DS and WW roots (Figure 6). The data shown in Figure 2 is consistent with the observation that the transcript populations in drought recovered and well-watered roots are far more similar than either is to the transcript population in drought-stressed roots. Taken together, these results suggest that the root transcriptome in *P. taeda *seedlings can return to its pre-drought state within 48 hours of recovery from the applied stress. Moreover, the drought-recovered plants (at harvest) no longer displayed the wilting phenotype seen in drought stressed plants and DR root tips had changed from the totally brown appearance seen in DS roots to having copious numbers of white root tips characteristic of control roots (data not shown).

Sixty-four of the 76 DE genes identified in the drought recovered samples were up-regulated and 30 of these had little sequence homology to known genes (E-value > 1 × 10^-5^). Significant overlap between drought-recovered and drought-stressed expression patterns was seen as 71 of 76 genes identified in DR roots were also identified in DS roots (Figure 6). These included multiple genes encoding apparent LEAs, chitinases, and β-glucosidases (Additional File 7). Possible regulatory genes identified in the drought-recovered sample include a two-component histidine-kinase response regulator protein (11.1.17.15), an ethylene-responsive RNA helicase (8.2.16.16), a gibberellin-regulated protein (10.3.10.14), and TFs belonging to the WRKY (7.3.15.5) and zinc finger (9.3.15.22) classes. With the exception of the gibberellin-regulated protein, putative regulatory genes identified in drought-recovered roots were also found in the drought-stressed roots where they all demonstrated increased expression. Few genes showed reduced expression after drought recovery and only one, an α-pinene synthase, showed expression reduced > 2-fold. Among the genes down-regulated in drought-recovered roots were ones thought to encode pinene synthases, a pectate lyase, a xyloglucan endo-transglycosylase, and a β-glucosidase. Only five DE genes were identified exclusively in drought recovered roots. These appeared to encode a ribosomal protein (11.3.20.24), a xyloglucan endo-transglycosylase (11.2.17.17), a gibberellin-regulated protein (10.3.10.14), and two unknowns.

## Discussion

### PtGen2 Microarray

The loblolly pine microarray (PtGen2) that is described here contains 26,496 features, including 25,848 distinct cDNAs, making it the largest publicly available microarray for any conifer species. PtGen2 can also be used for gene expression studies in species other than *P. taeda*. For example, it has been used to evaluate transcriptome dynamics in staged embryos from *P. pinaster *[62], and to compare expression profiles from several closely related pine and conifer species (data not shown). The data presented here demonstrate the utility of PtGen2 in the evaluation of transcriptome responses in loblolly pine and identifies classes of genes whose expression profiles are consistent with results from previous drought studies in conifers and angiosperms. The cDNA sequences printed on PtGen2, as well as the contigs to which they belong, can be accessed at the Fungen website [35]. PtGen2 chips, detailed protocols, complete annotation files, and the .gal file can be obtained by contacting the authors.

### Molecular Basis of Drought Tolerance in Pine Roots

Root transcriptome responses to drought stress were characterized by 9.6% of the genes interrogated with PtGen2 being differentially expressed by at least 1.5-fold. Forty-nine percent of DE genes were up-regulated and 51% were down-regulated, indicating that down-regulation of transcripts is likely a non-trivial aspect of the drought response in *P. taeda *roots. A similar percentage of DE genes (12.5%) were also observed in *P. taeda *needles after cycles of mild and severe drought stress, and an approximately equal number of transcripts were both up- and down-regulated during the early stages of mild and severe drought stress [29]. Analyses of transcriptome responses to water stress in angiosperms, e.g. *Arabidopsis *[63,64], rice [65], barley [66,67], and grape [68] identified 4-16% of the genes interrogated as being differentially expressed. This is in stark contrast to *Populus *subjected to gradual drought stress where only 1.5% of all genes and < 1% of root-responsive genes were differentially expressed at a ≥ 2-fold level [26]. Using a 2-fold cut-off for our data, the percentage of DE genes in drought-stressed pine roots is reduced to 3.5% which is still significantly higher than the *Populus *value. A complete explanation for this difference remains to be elucidated.

The complexity of transcriptome responses to drought stress makes comparisons among the number and type of DE genes identified in previous studies difficult as some differences are no doubt due to the duration and severity of stress, the tissue under study, the experimental design, and the statistical treatment of data. Nevertheless, the differential expression patterns seen for specific classes of genes in this study were generally consistent with what has been documented in other plant species subjected to severe water or salt stress. Many of the drought stress responsive genes found in this study, whether identified by the number of probes representing them, their log_2 _values, or by gene network analysis, have previously been well documented in angiosperm drought responses. This suggests evolutionary conservation of the molecular mechanisms utilized by both gymnosperms and angiosperms for response to water deficits. In drought-stressed roots, the majority of DE genes displaying increased transcript abundance fall into the tolerance or protective category of drought-responsive genes, probably because the root samples were so severely stressed at the time of harvest that genes involved in early sensory and regulatory responses were underrepresented.

Numerous studies have addressed the physiological recovery from drought stress in pines and conifers [69-72], but very little information is available on transcriptome responses during drought recovery. What is clear from this study is that the transcript population in drought-stressed loblolly roots can return to nearly pre-drought transcript population levels within 48 hours of removing the stress (Figures 2 and 6). Of 76 DE genes identified in DR roots, 93% were found to be expressed at elevated levels in DS roots. One gene that stood out in drought recovery was a gibberellic acid-binding protein. However, we did not see differential expression in any gibberellic acid 2-oxidase genes, even though these genes have frequently been reported as induced by stress conditions, including drought. Gibberellic acid signaling could have relevance for drought tolerance through its effect on plant morphology. Future studies that include multiple time points to more precisely pinpoint when drought recovery begins and how long it takes to complete would help identification of transcriptome changes that occur early in the recovery process.

### Candidate Protection Genes

A large gene family commonly seen in drought response studies is the late embryogenesis abundant (LEA) proteins, which includes dehydrins [73]. Their precise function(s) is unknown, but they are believed to be involved in a number of protective processes, including acting as hydration buffers, antioxidants, metal ion binding proteins, and both enzyme and membrane stabilizers (reviewed) [74]. Studies have shown the induction of LEA and dehydrin transcripts, as well as increased production of the encoded proteins, in response to osmotic stress [74,75]. LEA gene expression was previously shown to be up-regulated in pine shoot and stem tissues responding to drought stress [28,29]. Twenty-six of the 31 probes on the PtGen2 microarray that appear to encode LEAs or dehydrins showed their genes to be up-regulated in DS roots. Three of these (3.1.11.9, 2.4.16.12, and 1.3.16.12) were highly induced and found to be highly ranked in the gene network analysis. This supports the belief that LEAs play an important role in mitigating the effects of drought stress and also demonstrates the identification of an expected class of drought-responsive genes by PtGen2. These results also suggest that involvement LEA gene function in drought tolerance is conserved among seed plants.

Compatible solutes (osmolytes) are small solutes (primarily sugars and amino acids, such as proline) that are thought to protect cells by maintaining turgor pressure and stabilizing proteins and membranes under osmotic-stress conditions [76,77]. Sugar-based osmolytes, such as sucrose and raffinose family oligosaccharides (RFOs), are thought to be particularly important for reducing the effects of osmotic stress in plants [77,78]. DE genes thought to encode sucrose synthase, galactinol synthase, and raffinose synthase were up-regulated in drought stressed roots. Genes thought to be involved in proline synthesis also were up-regulated, while almost all genes identified as "proline-rich protein" were down-regulated.

Desiccation-tolerant plants accumulate high concentrations of sucrose during dehydration which, in conjunction with LEAs and dehydrins, may act to stabilize drying cells through direct interactions with macromolecules and membranes [78]. In maize ear and tassel tissues, *Sus *transcripts increased during water deficit stress [79]. In *A. scabra*, a thermotolerant grass species, increased accumulation of sucrose synthase in the roots was proposed to contribute to superior root thermotolerance by regulating sucrose metabolism [80]. Analysis of differentially expressed ESTs has shown increased *Sus *transcript levels as well as elevated of sucrose levels in water-stressed citrus seedlings [81]. Interestingly, *Sus *transcript levels were induced in water-stressed *Populus *leaves, but were consistently repressed in roots during the early stages of water deficit [26]. Up-regulation of *Sus *gene transcripts in water-stressed pine roots may be related to either the generation of sucrose or to the regulation of hexose flux. For example, both white spruce (*P. glauca*) and jack pine (*P. banksiana*) seedlings accumulated higher concentrations of sucrose in roots relative to stems after exposure to elevated salt concentrations [82], while in *Arabidopsis*, sucrose synthase was shown to control intracellular glucose levels and transitory starch biosynthesis [83]. Similar alterations in sugar metabolism likely occur in pine roots as all genes on the PtGen2 microarray encoding putative invertases and hexokinases were up-regulated under drought stress. One suggested regulatory mechanism for SuS is that its role in sucrose cycling impacts flux through the hexokinase reaction [84] Meanwhile, both hexokinases and cell wall invertases are thought to play key regulatory roles in assimilate partitioning during stress [85,86].

Up-regulation of *SuS *genes could also be related to raffinose family oligosaccharide (RFO) biosynthesis. Galactinol synthase catalyzes the first committed step in RFO synthesis, utilizing sucrose and galactinol as substrates and, thus, plays a regulatory role in carbon partitioning [87]. In *Arabidopsis*, it was shown that *GolS1 *is responsible for heat stress-dependent synthesis of raffinose [88], while both *GolS1 *and *GolS2 *are up-regulated in response to drought treatment. Over-expression of *GolS2 *in transgenic *Arabidopsis *leads to improved drought tolerance [89]. It also has been suggested that the osmoprotectant ability of galactinol and raffinose is due to their participation in ROS scavenging, because both can competitively inhibit hydroxyl radical-induced formation of 2,3-dihydroxy-benzoic acid (DHBA) *in vitro *[90].

An inositol transporter gene (3.2.1.7) was highly ranked in the network analysis, which could suggest a role in osmoprotection. *Myo*-inositol is a substrate for conjugation with galactose to form galactinol. Accumulation of *myo*-inositol and its methylated derivatives is correlated with drought tolerance [91], and it has been hypothesized that increased transport of inositols and its derivatives may act to counter osmotic stress [92]. The large number of up-regulated DE genes related to the biosynthesis and regulation of osmolytes in conjunction with an inositol transporter hub gene strongly suggests an important role for osmoprotectants in the response of pine roots to drought stress. Physiological and biochemical studies will be needed to address this hypothesis more thoroughly.

Aquaporins (AQPs) are members of the major intrinsic protein (MIP) family involved in increasing water diffusion across membranes by raising hydraulic or osmotic permeability [93,94]. Disparate expression patterns for these proteins in response to water deficit is common and this, in conjunction with the large number of AQP genes typically found in plants as well as their highly variable tissue-specific expression patterns, have confounded attempts to determine their specific roles in drought response [28,95-98]. Transgenic plants that over-express a class of AQPs called plasma membrane intrinsic proteins (PIPs) have shown both increased and decreased drought tolerance [99,100]. In this study, 17 of 18 probes for pine AQPs responded to drought stress. Most fell into the PIP category, and 16 were down-regulated (one *Arabidopsis *PIP2-6 ortholog was up-regulated). In *Arabidopsis *subjected to gradual drought stress, AQPs were generally down-regulated [101], as was the case for PIPs identified in tobacco [102] and *Populus *roots [26]. Thus, down-regulation of AQPs in drought-stressed pine roots appears to follow a pattern similar to that seen in some angiosperms and may reflect a mechanism for water conservation via reduced membrane permeability that minimizes water efflux into the surrounding soil [95].

### Candidate Regulatory Genes

Apparent transcription factors (TFs) from classes known to play a role in drought and abiotic stress responses in plants via ABA-dependent and ABA-independent mechanisms were differentially expressed in water-stressed pine roots. Genes encoding putative DREB1, bZIP, AP2/ERF, MYB, NAC, and WRKY TFs were all up-regulated. An apparent WRKY TF displayed the highest log_2 _ratio of any TF identified in DS roots. Interestingly, one of the most common drought response regulatory genes (DREB2) was not seen among the DE genes in either DS or DR roots. On the other hand, DREB1 TFs are more often associated with cold stress in plants [1]. Whether the apparent DREB1 identified in water-stressed pine roots plays a similar role to DREB2 in other plants remains to be determined.

Correlation network analysis did not identify any of the up-regulated TFs in water-stressed pine roots as hub nodes. However, network analysis did identify three up-regulated DE genes that may have a regulatory role in pine root responses to drought stress, cardiolipin synthase/phosphatidyltransferase, 9-cis-epoxycarotenoid dioxygenase, and zeatin o-glucosyltransferase, which could influence phospholipid metabolism, ABA synthesis, and cytokinin availability, respectively. The most likely explanation for why "classic" regulatory TFs, such as DREB2 and bZIP, were not seen as highly ranked network hubs in this study is a matter of experimental design. By the time water-stressed roots were harvested they were so severely stressed that transcription network dynamics may have progressed to a point where genes were more likely to be involved in stress mediation than to response of the onset of water deficiency. This would explain why the majority of up-regulated hubs listed in Table 3 identified genes encoding proteins involved in protection from ROS-mediated cell death (thioredoxin, peroxidase, metallothionein), transport (inositol transporter 2), cell/macromolecule protection (LEA, dehydrin, HSP70), and defense (NBS-LRR, phenylcoumaran benzylic ether reductase).

We identified an apparent WRKY gene up-regulated in both drought stress and drought recovery that is a candidate transcription factor for the regulation of drought response in pine roots. WRKYs comprise a large family of conserved plant TFs that are up-regulated in response to wounding, infection, and abiotic stress and that play multiple roles in developmental and metabolic pathways [103,104]. In *Arabidopsis *and rice, co-regulatory networks have been identified for WRKY genes involved in both defense and osmotic stress responses [105]. WRKY TFs induced by drought stress have been identified in rice and pennycress [106-108], while an *Arabidopsis *WRKY TF has been shown to play a critical role in both ABA response and drought tolerance [109]. Drought tolerance has been conferred on transgenic *Arabidopsis *expressing rice and soybean WRKY genes [107,110]. Recently, a WRKY TF was shown to participate in dehydration tolerance by binding to W-box elements of the galactinol synthase promoter in *Boea hygrometrica *[111]. Given that all galactinol synthase genes showed increased transcript abundance in drought-stressed samples, we can speculate that their expression in pine roots may reflect regulation by a WRKY TF.

ABA is considered something of a universal stress hormone in higher plants and it plays a central role in the regulation of responses to abiotic stress [112]. Although the primary site for ABA synthesis is generally considered to be the roots (from which it is released to the xylem for translocation to the rest of the plant), recent studies have suggested that ABA synthesis may also occur in shoot tissues [113]. Both ABA-dependent and -independent signaling pathways have been described in stress responses to drought, salinity and cold [1]. The gene 9-cis-epoxycarotenoid dioxygenase (NCED) catalyzes the first dedicated step and is the key enzyme in ABA biosynthesis [114]. In transgenic *Arabidopsis*, an NCED gene was shown to increase endogenous ABA levels resulting in enhanced drought tolerance [115]. In legumes and shrub species undergoing water-deficit induced stress, elevated NCED gene expression has been correlated with concomitant increases in ABA [116,117]. A gene thought to encode NCED was up-regulated in drought stressed pine roots and ranked high as a network hub. A second hub gene related to ABA signaling (ABA-responsive protein) was also identified from the network analysis. These observations are consistent with the regulatory role that NCEDs are thought to play in response to water stress in angiosperms and makes NCED a logical choice for having a similar function in pine roots.

Other translocated factors, such as cytokinins, may also originate in roots and modulate ABA signaling to impact shoot responses to drought [6,10]. The importance of cytokinins in drought-induced changes of root morphology has been suggested based on their accumulation in drought-stressed tobacco roots [118]. In this study, a gene encoding an apparent zeatin-O-glucosyl transferase (ZOG) was strongly up-regulated and was identified as a potential network regulator, while a second ZOG and a cytokinin oxidase displayed moderate increases in transcript abundance. Over-expression of *ZOG*1 in transgenic tobacco resulted in increased total cytokinin content, although bioactive cytokinins did not change significantly [119]. O-glucosylated forms of *trans*-zeatin are protected from oxidases and dehydrogenases and may act as cytokinin storage forms that can be converted quickly to bioactive cytokinins by the action of β-glucosidases [120]. Reducing bioactive cytokinin availability via glucosylation may be a regulatory mechanism for lowering the cytokinin:ABA ratio in pine roots to lessen the stimulatory effect of cytokinins on root meristematic tissue.

Correlation network analysis is a powerful method for mining complex "omics" datasets and the evaluation of gene networks often leads to identification of novel genes involved in particular processes or pathways, as well as identification of putative regulatory genes. In plants, this approach has seen increasing use, particularly in model species. For example, a genome-wide co-expression network in *Arabidopsis *identified 127 functional modules, fourteen of which were associated with stress and defense [121], and correlation networks in *Arabidopsis *[122] and rice [123] have identified co-expression modules that allude to the involvement of DREB genes in the regulation of trehalose-6-phosphate. However, few studies prior to this have specifically targeted drought responses.

In the current study, a variety of apparent transcription factors of classes known to regulate drought and water-deficit responses in other plants were found among DE genes in pine roots; however, none of these ranked highly in the network analysis. The most highly ranked network hub gene, although it is of unknown function, is of great interest. Other highly ranked hub genes appear related to protective functions rather than regulatory roles. Nevertheless, the apparent NCED and ZOG genes identified in this study have not been reported previously to respond to drought stress in pines. The crucial role these genes likely play in the ABA and cytokinin signaling pathways, respectively, should make them foci for future molecular investigations of water-stress responses in roots.

## Conclusion

A 26,496 feature microarray, PtGen2, fabricated for use in loblolly pine and conifer transcriptomic studies was used to identify 2445 DE genes (mean log_2 _ratio ≥ 1.5, FDR ≤ 0.01) in DS roots and 76 DE genes in DR roots after a 48 hour recovery period. Differential gene expression returned to a near normal pattern within 48 hours of drought stress recovery indicating that transcriptome recovery from severe drought is a fairly rapid process in pine roots. Approximately 30% of DE genes identified in drought-stressed roots and 25% of the up-regulated hub nodes identified by weighted gene correlation network analysis had no significant homology to any known or identifiable genes (E-value ≥ 1 × 10^-5^), demonstrating the need for continued gene identification and annotation efforts in this species. Coordinated gene expression patterns, along with gene network analysis, helped to identify a number of candidate genes that are likely involved in both protective and regulatory capacities in pine root water stress responses. To our knowledge, this is the largest conifer array yet produced and the first example of correlation gene network analysis being used to assess transcriptional responses to drought in any tree species. This study clearly demonstrates the utility of the PtGen2 microarray for evaluating transcriptome dynamics in pine and has identified numerous genes that will be the subject of future study as we expand our understanding of drought responses in this important commercial tree species.

## Authors' contributions

**WL **directed fabrication of the microarray, oversaw and developed protocols and participated in all aspects of microarray processing, data collection, generation of probe annotations, statistical analysis, and writing and editing of the manuscript. **RA **participated in statistical and network analysis of the data, contributed to data interpretation, provided important intellectual input, and contributed to writing and editing the manuscript. **YY **performed sample processing and labeling, microarray hybridizations, scanning, data processing, and participated in probe annotation efforts. **JB **was involved in cDNA library maintenance, sample amplification, and fabrication of the array. **MS **performed sample processing and labeling, microarray hybridizations, scanning, and data processing. **JD **was involved in microarray design, experimental design, providing critical intellectual content, and was involved in writing and editing the manuscript. All authors have read and approved the final manuscript.

## Supplementary Material

Additional file 1 **Microarray reference sample composition**. This file shows the composition of the microarray reference sample used in all hybridizations. And includes information regarding the tissue, maternal genotype, treatment, tissue origin, and mass of total RNA used to make the reference standard. NA = not applicable, WW = well-watered, DS = drought stressed, DR = drought plus 48 hr. recovery. Click here for file

Additional file 2 **Primer pair sets used in the RT-qPCR analysis**. This file contains the sequences of primer pairs used in qPCR analysis. Gene ID = unique probe address (metarow, metacolumn, row, column), UniScript = Fungen assembly contig ID. Click here for file

Additional file 3 **This Excel file contains the complete PtGen2 master file including annotations and print tip information (3A), the list of duplicate spotted validation cDNAs and buffer blanks that were excluded from statistical analysis (3B), and 251 probes removed from statistical analysis since they mapped to contigs already represented by other cDNAs**. 3A (PtGen2 Master Annotation File): Gene ID = unique probe address (metarow, metacolumn, row, column), UniScript = Fungen assembly contig ID, Clone_Name = cDNA clone ID. P_C_R actual = plate, column, and row or source print plates. P_C_R Virtual = plate_column_row with virtual plate 69 (actually a reprint of plate 10). Print Tip = print tip location on 48 pin head. All BlastX results are ordered by database name abbreviation followed by accession (BlastX), description (ID), and E-value (Exp). NCBI = NCBI non-redundant, SP = Swiss Prot, TR = TrEMBL, UREF = UniRef100, TAIR = Arabidopsis, MT2P = Medicago, OSA = rice. **3B (Plate 69 + Buffers) **Gene IDs and clone names for duplicate spotted plate 69 (re-spot of source plate 10) used only for validation purposes and removed from statistical evaluation of experimental data. A correlation analysis was done on these 384 probes (see Additional File 5). **3C (251 Duplicate cDNAs)**: Gene IDs and clone names of 251 cDNAs removed from statistical evaluation of experimental data since these clones were duplicate probes that derived from cDNA clones that mapped to the same Uniscript (contig). Click here for file

Additional file 4 **R-script code for WGCNA**. This file contains the modified R-script code used to perform WGCNA for generation of the loblolly pine root transcriptome gene network. Click here for file

Additional file 5 **R-correlation analysis of 384 replicated probes**. This file contains the results of an R-correlation analysis of BRB filtered log_2 _ratios for a set of 384 replicate probes on PtGen2. Sample ID = CCLONE genotype identifier followed by treatment, WW = well-watered, DS = drought-stressed, DR = drought plus 48 hr recovery. Six paired hybridizations were preformed and each paired hybridization was performed with identical target samples, i.e., replicates 1, 2, and 3 are correlations for the averages of paired hybridizations for the set of 384 replicate probes. The average of the three replicates was calculated to give an R-value for each of the 12 samples. Click here for file

Additional file 6 **Quantitative PCR analysis of highly differentially expressed genes and moderately expressed transcription factors**. This file contains the results of qPCR analysis performed on 16 genes that were used to compare fold expression levels with those determined using PtGen2. RT-qPCR values were corrected to Actin 1 gene for each sample. For each treatment group three qPCR measurements were taken for each of four biological replicates and then averaged. Gene ID = the physical address of the spot on the array (metarow, metacolumn, row, column), UniScript = Fungen assembly contig ID. Microarray = log_2 _mean ratios and RT-qPCR = absolute fold difference after normalization to the *P. taeda *actin2 gene. Click here for file

Additional file 7 **Expression Results for differentially expressed genes (DEGs) identified after interrogation of PtGen2**. This Excel file contains three separate sheets that list the normalized log_2 _mean ratios for differentially expressed genes exhibiting at least a 1.5-fold difference obtained after pair wise comparisons for each of the three water availability treatments (drought stressed (DS), well-watered (WW), and drought recovered (DR). Note that each table can be sorted by either fold-increase or fold-decrease expression to coincide with results and discussion sections. **7A **(DS versus WW) 2445 DEGs with at least 1.5 fold expression difference identified in the DS versus well-WW comparison. **7B **(DR versus WW) 76 DEGs with at least 1.5 fold expression difference identified in the DR versus WW comparison. **7C **(DS versus DR) 1918 DEGs with at least a 1.5-fold expression difference identified in the DS versus DR comparison. All three tables show Gene ID = the physical address of the spot on the array (metarow, metacolumn, row, column), UniScript = FUNGEN contig ID, Clone_Name = cDNA clone ID, Fold ↑, and Fold ↓values are displayed as normalized log_2 _mean ratios. All BlastX results are ordered by database name abbreviation followed by accession (BlastX), description (ID), and E-value (Exp). NCBI = NCBI non-redundant, SP = SwissProt. Click here for file

Additional file 8 **Gene correlation analysis and module membership data generated using WGCNA**. This file contains the WGCNA module memberships for the 2445 differentially expressed genes identified in the drought stressed versus well-watered analysis as well as log_2 _mean ratios and BlastX annotation information. Each module color represents a grouping of genes whose expression profiles are coordinately expressed. Module membership correlation values were used to generate the graphical representation of the pine root gene network. Gene ID = the physical address of the spot on the array (metarow, metacolumn, row, column), Fold ↑, and Fold ↓values for DS vs. WW comparison are displayed as normalized log_2 _mean ratios. Module color, MM = module membership correlation value, p.MM = probability of module membership. All BlastX results are ordered by database name abbreviation followed by accession (BlastX), description (ID), and E-value (Exp). NCBI = NCBI non-redundant, SP = Swiss Prot. Click here for file
