# Supplementary material for: Microarray analysis and scale-free gene networks identify candidate regulators in drought-stressed roots of loblolly pine (P. taeda L.)
Source: BMC Genomics. 2011 May 24;12:264. doi: 10.1186/1471-2164-12-264 (PMC3123330; doi:10.1186/1471-2164-12-264)
Supplement: Additional file 1 — Microarray reference sample composition. This file shows the composition of the microarray reference sample used in all hybridizations. And includes information regarding the tissue, maternal genotype, treatment, tissue origin, and mass of total RNA used to make the reference standard. NA = not applicable, WW = well-watered, DS = drought stressed, DR = drought plus 48 hr. recovery. [file 1471-2164-12-264-S1.PDF]

Additional File 1

Sources for total RNA used in microarray reference standard

***P.taeda* Genotype**	**Treatment**	**Tissue**	**Mass**
CClone 40430	NA	Candle, Needle	500ug
CClone 43680	NA	Candle, Needle	500ug
CClone 41586	NA	Candle, Needle	500ug
CClone 41201	WW	Root	250ug
CClone 41201	DS	Root	250ug
CClone 41201	DR	Root	250ug
CClone 41396	WW	Root	250ug
CClone 41396	DS	Root	250ug
CClone 41396	DR	Root	250ug
CClone 44686	WW	Root	250ug
CClone 44686	DS	Root	250ug
CClone 44686	DR	Root	250ug
CClone 45226	WW	Root	250ug
CClone 45226	DS	Root	250ug
CClone 45226	DR	Root	250ug
7-56	NA	Callus (needle origin)	250ug
