# Supplementary material for: Microarray analysis and scale-free gene networks identify candidate regulators in drought-stressed roots of loblolly pine (P. taeda L.)
Source: BMC Genomics. 2011 May 24;12:264. doi: 10.1186/1471-2164-12-264 (PMC3123330; doi:10.1186/1471-2164-12-264)
Supplement: Additional file 2 — Primer pair sets used in the RT-qPCR analysis. This file contains the sequences of primer pairs used in qPCR analysis. Gene ID = unique probe address (metarow, metacolumn, row, column), UniScript = Fungen assembly contig ID. [file 1471-2164-12-264-S2.PDF]

Additional File 2

Primer pairs used in RT-qPCR Analysis

Gene ID	Uniscript	Left Primer	Right Primer
10.2.21.6	2_3931	CTGACGCGGTCCTTAACTTC	TCAAGCAATCAATGGAGCAG
11.2.16.23	2_10347	ATCCAGCAAGAAGAGGACCA	CTGCGTGTGCTTGACATCTT
1.3.16.12	2_5201	TAAAGATGAGCGTCCGGAGT	AGCAGGGCGATCACATTAAG
4.1.22.8	2_8298	TTCGGTGTCCTCATCACAGA	TGAAGATGCTGCCTAAAGCA
3.2.8.7	2_6459	TTCCACCATTACAGCGTTGA	CTGCCTGTGTCTCCAACTTG
7.2.16.23	2_8048	ATTTCCAGAAGTGCCAGGTG	GCCCTGCAACAATCTCTAGC
7.2.16.5	2_8167	CTGCACAGAAGACCACGAAA	CGTGGAATTAATGGGAACGA
10.1.13.7	0_5245	GGCAGGACTGACAACGAAAT	CGCAGTGAGCTTTCTGAATG
11.3.19.21	2_32	ATGCTCCGAATCAACTCCAC	GTCAGGTTCGTTGGTCAGCT
5.2.15.14	2_3847	ACCGTGGTGTCTCTTGGTTC	GGACCGACTTTGATGCAAAT
2.1.7.11	0_12961	CAATGAGAGATGCCCGAAAT	TTGCTCTGCCATTCAAACAG
11.3.19.9	2_8497	CGTCGCTTCTTGTAGGGTTC	GCACAGATTCGACTCAAGCA
7.3.19.20	2_2267	GGAGAAACCTGCATCGCTAC	ATTCTGGAGGCAAGGAACG
12.1.21.9	2_2508	CAAGATGTGAACCCAGTCGA	GTGCGTGGTGTTGTAGCATC
1.2.18.1	2_9495	TGCTGAAACAAACTGGTTGC	GGCCTTGGTATGATGTCTGG
11.4.5.8	0_14551	TTACAAGGGAAGGCAGCTTC	AAACACCGAATTGCAGATCC
NA	P.t._ACT2	TTGCTGACCGTATGAGCAAG	GAGGTGCAACCACCTTGATT
