# Supplementary material for: Microarray analysis and scale-free gene networks identify candidate regulators in drought-stressed roots of loblolly pine (P. taeda L.)
Source: BMC Genomics. 2011 May 24;12:264. doi: 10.1186/1471-2164-12-264 (PMC3123330; doi:10.1186/1471-2164-12-264)
Supplement: Additional file 4 — R-script code for WGCNA. This file contains the modified R-script code used to perform WGCNA for generation of the loblolly pine root transcriptome gene network. [file 1471-2164-12-264-S4.PDF]

Additional File 4

R-correlation analysis of hybridization results for 384 replicated probes on PtGen2

Sample ID	Replicate 1	Replicate 2	Replicate 3	AVG
41201_WW	0.785	0.711	0.532	0.676
41369_WW	0.835	0.696	0.618	0.716
44686_WW	0.828	0.745	0.772	0.782
45226_WW	0.850	0.788	0.723	0.787
41201_DS	0.922	0.822	0.696	0.814
41369_DS	0.852	0.839	0.805	0.832
44686_DS	0.884	0.753	0.860	0.832
45226_DS	0.747	0.747	0.779	0.757
41201_DR	0.721	0.769	0.734	0.741
41369_DR	0.805	0.792	0.814	0.804
44686_DR	0.828	0.832	0.831	0.830
45226_DR	0.826	0.739	0.683	0.749
