# Supplementary material for: Microarray analysis and scale-free gene networks identify candidate regulators in drought-stressed roots of loblolly pine (P. taeda L.)
Source: BMC Genomics. 2011 May 24;12:264. doi: 10.1186/1471-2164-12-264 (PMC3123330; doi:10.1186/1471-2164-12-264)
Supplement: Additional file 5 — R-correlation analysis of 384 replicated probes. This file contains the results of an R-correlation analysis of BRB filtered log2 ratios for a set of 384 replicate probes on PtGen2. Sample ID = CCLONE genotype identifier followed by treatment, WW = well-watered, DS = drought-stressed, DR = drought plus 48 hr recovery. Six paired hybridizations were preformed and each paired hybridization was performed with identical target samples, i.e., replicates 1, 2, and 3 are correlations for the averages of paired hybridizations for the set of 384 replicate probes. The average of the three replicates was calculated to give an R-value for each of the 12 samples. [file 1471-2164-12-264-S5.DOC]

**#Here are some things that must be installed in R before this script will run. First, open R and install the # following R packages with this command:**

install.packages(c("fields", "impute", "dynamicTreeCut", "qvalue", "flashClust", "Hmisc") )

**#Next, find the latest WGCNA package, currently 0.85. It may be in the package file pull down menu, #If not, download the zip file from the WGCNA web site and install package from the zip file**

**#(R has a command to do this). Now you’re ready to process your data. Cut and paste each #commented (#) section to go through the whole process.**

**#PART 1: Loading and Cleaning the Data**

library(WGCNA)

setwd("C:/Pine_Gene_Network/ModuleRun_2-1-10")

options(stringsAsFactors = FALSE);

**#Read in the data set**

dat1=read.csv("ExprDataWWDSDR_2445impute.csv", header=T)

**#Take a quick look at what is in the data set**

dim(dat1)

names(dat1)

datExpr0 = as.data.frame(t(dat1[, -c(1:10)]));

names(datExpr0) = dat1$Gene_ID;

rownames(datExpr0) = names(dat1)[-c(1:10)];

**#Cluster the samples to inspect for outlier arrays. Plot the sample tree: Open a graphic output window of size #12 by 9 inches. The user should change the dimensions if the window is too large or too small**

sampleTree = flashClust(dist(datExpr0), method = "average");

sizeGrWindow(12,9)

par(cex = 0.6);

par(mar = c(0,4,2,0))

plot(sampleTree, main = "Sample clustering to detect outliers", sub="", xlab="", cex.lab = 1.5,cex.axis = 1.5, cex.main = 2)

**#Plot a line to show the cut (can trim outliers)**

abline(h = 90, col = "red");

**#Determine cluster under the line**

clust = cutreeStatic(sampleTree, cutHeight = 90, minSize = 10)

table(clust)

**#clust 1 contains the samples we want to keep**

keepSamples = (clust==1)

datExpr = datExpr0[keepSamples, ]

nGenes = ncol(datExpr)

nSamples = nrow(datExpr)

**#Load trait data**

traitData = read.csv("TraitDataWWDSDR_2445.csv");

dim(traitData)

names(traitData)

**#Remove columns that hold information we do not need**

allTraits = traitData[, -c(5, 4)];

allTraits = allTraits[, c(2, 3:4)]

dim(allTraits)

names(allTraits)

**#Form a data frame analogous to expression data that will hold the clinical traits**

WWDSDRSamples = rownames(datExpr);

traitRows = match(WWDSDRSamples, allTraits$Stage);

datTraits = allTraits[traitRows, -1];

rownames(datTraits) = allTraits[traitRows, 1];

collectGarbage();

**#Re-cluster the samples**

sampleTree2 = flashClust(dist(datExpr), method = "average")

**#Convert traits to a color representation; white means low, red means high, grey means missing**

traitColors = numbers2colors(datTraits, signed = FALSE);

**#Plot the sample dendrogram and the colors underneath.**

plotDendroAndColors(sampleTree2, traitColors, groupLabels = names(datTraits), main = "Sample dendrogram and trait heatmap")

**#Save the relevant expression and trait data for use in the next steps.**

save(datExpr, datTraits, file = "WWDSDR_2445-01-dataInput.RData")

**#PART2: Network Construction and Module Detection; load the data saved above (load the variables #datExpr and datTraits containing the expression and trait data)**

lnames = load(file = "WWDSDR_2445-01-dataInput.RData");

**#The variable lnames contains the names of loaded variables.**

lnames

**#Choose the soft-thresholding power for analysis of network topology**

powers = c(c(1:10), seq(from = 12, to=20, by=2))

**#Call the network topology analysis function**

sft = pickSoftThreshold(datExpr, powerVector = powers, verbose = 5)

**#Plot the results; scale-free topology fit index as a function of the soft-thresholding power and mean connectivity as a function of the soft-thresholding power**

sizeGrWindow(9, 5)

par(mfrow = c(1,2));

cex1 = 0.9;

plot(sft$fitIndices[,1], -sign(sft$fitIndices[,3])*sft$fitIndices[,2], xlab="Soft Threshold (power)",ylab="Scale Free Topology Model Fit, signed R^2", type="n", main = paste("Scale independence"));

text(sft$fitIndices[,1], -sign(sft$fitIndices[,3])*sft$fitIndices[,2], labels=powers,cex=cex1,col="red");

abline(h=0.90,col="red")

plot(sft$fitIndices[,1], sft$fitIndices[,5], xlab="Soft Threshold (power)",ylab="Mean Connectivity", type="n", main = paste("Mean connectivity"))

text(sft$fitIndices[,1], sft$fitIndices[,5], labels=powers, cex=cex1,col="green")

**#Now calculate the adjacencies using the soft thresholding power (i.e., softPower = beta)**

softPower = 16;

adjacency = adjacency(datExpr, power = softPower);

**#Turn adjacency matrix into topological overlap matrix; then convert to dissimilarity matrix (1-TOM).**

TOM = TOMsimilarity(adjacency);

dissTOM = 1-TOM

**#Call the hierarchical clustering function; flastClust is much faster clustering routine than hclust**

geneTree = flashClust(as.dist(dissTOM), method = "average");

**#Plot the resulting clustering tree (dendrogram)**

sizeGrWindow(12,9)

plot(geneTree, xlab="", sub="", main = "Gene clustering on TOM-based dissimilarity", labels = FALSE, hang = 0.04);

**#Module identification using dynamic tree cut**

minModuleSize = 30;

dynamicMods = cutreeDynamic(dendro = geneTree, distM = dissTOM, deepSplit = 2, cutHeight = 0.97, pamRespectsDendro = FALSE, minClusterSize = minModuleSize);

table(dynamicMods)

dynamicColors = labels2colors(dynamicMods)

table(dynamicColors)

**#Convert numeric labels into colors and plot the dendrogram and colors underneath**

sizeGrWindow(8,6)

plotDendroAndColors(geneTree, dynamicColors, "Dynamic Tree Cut", dendroLabels = FALSE, hang = 0.03,

addGuide = TRUE, guideHang = 0.05, main = "Gene dendrogram and module colors")

**#Dynamic Tree Cut may identify modules whose expression profiles are very similar; it may be prudent to #merge such modules since their genes are highly co-expressed; to quantify co-expression similarity of #entire modules calculate their eigengenes and cluster them on their correlation.**

**#Calculate eigengenes**

MEList = moduleEigengenes(datExpr, colors = dynamicColors)

MEs = MEList$eigengenes

**#Calculate dissimilarity of module eigengenes**

MEDiss = 1-cor(MEs);

**#Cluster module eigengenes**

METree = flashClust(as.dist(MEDiss), method = "average");

**#Plot the result**

sizeGrWindow(7, 6)

plot(METree, main = "Clustering of module eigengenes", xlab = "", sub = "")

**#To choose a height cut of 0.2 (corresponding to correlation of 0.8) and merge**

**#**This was the original setting from Rob, i.e. not 0.2, we used 0.025 for my modules

MEDissThres = 0.025

**#Plot the cut line into the dendrogram**

abline(h=MEDissThres, col = "red")

**#Call automatic merging function**

merge = mergeCloseModules(datExpr, dynamicColors, cutHeight = MEDissThres, verbose = 3)

**#The merged module colors**

mergedColors = merge$colors;

**#Eigengenes of the new merged modules**

mergedMEs = merge$newMEs;

**#To see what the merging did to our module colors, plot the gene dendrogram again, with the original and #merged module colors underneath**

sizeGrWindow(12, 9)

plotDendroAndColors(geneTree, cbind(dynamicColors, mergedColors), c("Dynamic Tree Cut", "Merged dynamic"),

dendroLabels = FALSE, hang = 0.03, addGuide = TRUE, guideHang = 0.05)

**#In the subsequent analysis, we will use the merged module colors in mergedColors; save the relevant #variables for use in subsequent parts of the tutorial; rename to moduleColors**

moduleColors = mergedColors

**#Construct numerical labels corresponding to the colors**

colorOrder = c("grey", standardColors(50));

moduleLabels = match(moduleColors, colorOrder)-1;

MEs = mergedMEs;

**#Save module colors and labels for use in subsequent parts**

save(MEs, moduleLabels, moduleColors, geneTree, file = "WWDSDR_2445-02-networkConstruction-stepByStep.RData")

**#PART3: Related modules to external information and identifying important genes**

**# Load the expression and trait data saved in the first part. The variable lnames contains the names of loaded #variables.**

lnames = load(file = "WWDSDR_2445-01-dataInput.RData");

lnames

**#Load network data saved in the second part.**

lnames = load(file = "WWDSDR_2445-02-networkConstruction-stepByStep.RData");

lnames

**#Since we have a summary profile (eigengene) for each module, correlate eigengenes with external traits and #look for the most significant associations**

**# Define numbers of genes and samples**

nGenes = ncol(datExpr);

nSamples = nrow(datExpr);

**#Recalculate MEs with color labels**

MEs0 = moduleEigengenes(datExpr, moduleColors)$eigengenes

MEs = orderMEs(MEs0)

moduleTraitCor = cor(MEs, datTraits, use = "p");

moduleTraitPvalue = corPvalueStudent(moduleTraitCor, nSamples);

**#Since we have a moderately large number of modules and traits, a graphical representation will help in #reading the table; we color code each association by the correlation value and display correlations and #their p-values within a heatmap plot**

sizeGrWindow(10,6)

textMatrix = paste(signif(moduleTraitCor, 2), "\n(", signif(moduleTraitPvalue, 1), ")", sep = "");

dim(textMatrix) = dim(moduleTraitCor)

par(mar = c(6, 8.5, 3, 3));

labeledHeatmap(Matrix = moduleTraitCor, xLabels = names(datTraits), yLabels = names(MEs), ySymbols = names(MEs), colorLabels = FALSE, colors = greenWhiteRed(50), textMatrix = textMatrix, setStdMargins = FALSE,

cex.text = 0.5, zlim = c(-1,1), main = paste("Module-trait relationships"))

table(dynamicColors)

**#Gene relationship to trait and important modules (i.e., gene significance and module membership). We #quantify associations of individual genes with our trait of interest by defining Gene Significance GS as the #absolute value of the correlation between the gene and the trait. For each module, we also define a #quantitative measure of module membership MM as the correlation of the module eigengene and the gene #expression profile. This allows us to quantify the similarity of all genes on the array to every module.**

**#Define variable Genotype as the trait from datTrait and define names (colors) of the modules**

Treatment = as.data.frame(datTraits$Treatment);

names(Treatment) = "Treatment"

modNames = substring(names(MEs), 3)

geneModuleMembership = as.data.frame(cor(datExpr, MEs, use = "p"));

MMPvalue = as.data.frame(corPvalueStudent(as.matrix(geneModuleMembership), nSamples));

names(geneModuleMembership) = paste("MM", modNames, sep="");

names(MMPvalue) = paste("p.MM", modNames, sep="");

geneTraitSignificance = as.data.frame(cor(datExpr, Treatment, use = "p"));

GSPvalue = as.data.frame(corPvalueStudent(as.matrix(geneTraitSignificance), nSamples));

names(geneTraitSignificance) = paste("GS.", names(Treatment), sep="");

names(GSPvalue) = paste("p.GS.", names(Treatment), sep="");

**#Intramodular analysis – identifying genes with high GS and MM. Using the GS and MM measures, identify #genes that have a high significance for Treatment as well as high module membership in interesting #modules. As an example, we look at the red module that has the highest association with Treatment. We #plot a scatterplot of Gene Significance vs. Module Membership in the blue module:**

module = "red"

column = match(module, modNames);

moduleGenes = moduleColors==module;

sizeGrWindow(7, 7);

par(mfrow = c(1,1));

verboseScatterplot(abs(geneModuleMembership[moduleGenes, column]), abs(geneTraitSignificance[moduleGenes, 1]), xlab = paste("Module Membership in", module, "module"), ylab = "Gene significance for Treatment", main = paste("Module membership vs. gene significance\n"), cex.main = 1.2, cex.lab = 1.2, cex.axis = 1.2, col = module)

**#Repeat above two steps for trait = Treatment and module = red**

Treatment = as.data.frame(datTraits$Treatment);

names(Treatment) = "Treatment"

modNames = substring(names(MEs), 3)

geneModuleMembership = as.data.frame(cor(datExpr, MEs, use = "p"));

MMPvalue = as.data.frame(corPvalueStudent(as.matrix(geneModuleMembership), nSamples));

names(geneModuleMembership) = paste("MM", modNames, sep="");

names(MMPvalue) = paste("p.MM", modNames, sep="");

geneTraitSignificance = as.data.frame(cor(datExpr, Treatment, use = "p"));

GSPvalue = as.data.frame(corPvalueStudent(as.matrix(geneTraitSignificance), nSamples));

names(geneTraitSignificance) = paste("GS.", names(Treatment), sep="");

names(GSPvalue) = paste("p.GS.", names(Treatment), sep="");

module = "pink"

column = match(module, modNames);

moduleGenes = moduleColors==module;

sizeGrWindow(7, 7);

par(mfrow = c(1,1));

verboseScatterplot(abs(geneModuleMembership[moduleGenes, column]), abs(geneTraitSignificance[moduleGenes, 1]), xlab = paste("Module Membership in", module, "module"), ylab = "Gene significance for Treatment", main = paste("Module membership vs. gene significance\n"), cex.main = 1.2, cex.lab = 1.2, cex.axis = 1.2, col = module)

Treatment = as.data.frame(datTraits$Treatment);

names(Treatment) = "Treatment"

modNames = substring(names(MEs), 3)

geneModuleMembership = as.data.frame(cor(datExpr, MEs, use = "p"));

MMPvalue = as.data.frame(corPvalueStudent(as.matrix(geneModuleMembership), nSamples));

names(geneModuleMembership) = paste("MM", modNames, sep="");

names(MMPvalue) = paste("p.MM", modNames, sep="");

geneTraitSignificance = as.data.frame(cor(datExpr, Treatment, use = "p"));

GSPvalue = as.data.frame(corPvalueStudent(as.matrix(geneTraitSignificance), nSamples));

names(geneTraitSignificance) = paste("GS.", names(Treatment), sep="");

names(GSPvalue) = paste("p.GS.", names(Treatment), sep="");

module = "green"

column = match(module, modNames);

moduleGenes = moduleColors==module;

sizeGrWindow(7, 7);

par(mfrow = c(1,1));

verboseScatterplot(abs(geneModuleMembership[moduleGenes, column]), abs(geneTraitSignificance[moduleGenes, 1]), xlab = paste("Module Membership in", module, "module"), ylab = "Gene significance for Treatment", main = paste("Module membership vs. gene significance\n"), cex.main = 1.2, cex.lab = 1.2, cex.axis = 1.2, col = module)

**#We have found modules with high association with our trait of interest, and have identified their central #players by the Module Membership measure. Now merge this statistical information with gene annotation #and write out a file that summarizes the most important results that can be viewed in Excel. The microarray #data are only annotated by probe ID names**

**#This command will return all probe IDs included in the analysis.**

names(datExpr)

**#This command will return all probe IDs included in the green, red, and pink modules.**

names(datExpr)[moduleColors=="green"]

names(datExpr)[moduleColors=="red"]

names(datExpr)[moduleColors=="pink"]

**#To facilitate interpretation of the results, use a probe annotation file to connect probe IDs to gene names #and universally recognized identification numbers.**

file = file(description = "GeneAnnotationDSDRWW.csv");

annot = read.csv(file = file);

dim(annot)

names(annot)

probes = names(datExpr)

probes2annot = match(probes, annot$Gene_ID)

**#The following is the number or probes without annotation (should return 0)**

sum(is.na(probes2annot))

**#We now create a data frame holding the following information for all probes: probe ID, gene symbol, Locus #Link ID, module color, gene significance for weight, and module membership and p-values in all modules. #The modules will be ordered by their significance for weight, with the most significant ones to the left.**

geneInfo0 = data.frame(Gene_ID = probes, UniScript = annot$UniScript[probes2annot], Func_Cat = annot$Func_Cat[probes2annot], CLONE_NAME = annot$CLONE_NAME[probes2annot], NCBI_Blastx = annot$NCBI_Blastx[probes2annot], NCBI_ID = annot$NCBI_ID[probes2annot], NCBI_Exp = annot$NCBI_Exp[probes2annot], UPSP_BlastX = annot$UPSP_BlastX[probes2annot], UPSP_ID = annot$UPSP_ID[probes2annot], UPSP_Exp = annot$UPSP_Exp[probes2annot], moduleColor = moduleColors, geneTraitSignificance, GSPvalue)

**#Order modules by their significance for weight**

modOrder = order(-abs(cor(MEs, Treatment, use = "p")));

**# Add module membership information in the chosen order**

for (mod in 1:ncol(geneModuleMembership))

{

oldNames = names(geneInfo0)

geneInfo0 = data.frame(geneInfo0, geneModuleMembership[, modOrder[mod]], MMPvalue[, modOrder[mod]]);

names(geneInfo0) = c(oldNames, paste("MM.", modNames[modOrder[mod]], sep=""), paste("p.MM.", modNames[modOrder[mod]], sep=""))

}

**#Order the genes in the geneInfo variable first by module color, then by gene Trait Significance**

geneOrder = order(geneInfo0$moduleColor, -abs(geneInfo0$GS.Treatment));

geneInfo = geneInfo0[geneOrder, ]

**#This data frame can be written into a text-format spreadsheet; Open and view in Excel)**

write.csv(geneInfo, file = "geneInfoWWDSDR_2445.csv")

**#PART3: Visualizing the network of eigengenes and specified traits**

**#It is often interesting to study the relationships among the found modules. Use the eigengenes as #representative profiles and quantify module similarity by eigengene correlation. The package contains the #function plotEigengeneNetworks that generates a summary plot of the eigengene network. It is usually #informative to add a clinical trait (or multiple traits) to the eigengenes to see how the traits fit into the #eigengene network:**

**#Recalculate module eigengenes**

MEs = moduleEigengenes(datExpr, moduleColors)$eigengenes

**#Isolate treatment of interest (drought) from the experimental traits. Not sure what this does**

Treatment = as.data.frame(datTraits$Treatment);

names(Treatment) = "Treatment"

**#Add the treatment trait to existing module eigengenes**

MET = orderMEs(cbind(MEs, Treatment))

**#Plot the relationships among the eigengenes and the trait. The function produces a dendrogram of the #eigengenes and trait(s), and a heatmap of their relationships. Dendro has treatment as a module???**

sizeGrWindow(5,7.5);

par(cex = 0.9)

plotEigengeneNetworks(MET, "", marDendro = c(0,4,1,2), marHeatmap = c(3,4,1,2))

**#PART4: Exporting to Cytoscape**

**#Cytoscape allows the user to input an edge file and a node file, allowing the user to specify for example the #link weights and the node colors. Here we demonstrate the output of two modules, the red and brown ones, #to Cytoscape.**

**#Recalculate topological overlap if needed.**

TOM = TOMsimilarityFromExpr(datExpr, power = 16);

**#Read in the annotation file and select modules (some of these steps may be redundant with above; delete?)**

file = file(description = "GeneAnnotationDSDRWW.csv");

annot = read.csv(file = file);

dim(annot)

names(annot)

table(dynamicColors)

**#**modules = c("black", "yellow", "greenyellow", "purple", "blue", "magenta", "brown", "turquoise", "pink", "green", "red")

**#**modules = c("pink", "green", "red")

**#**modules = c("red")

**#**modules = c("pink")

**#**modules = c("green")

**#Select module probes (some of these steps may be redundant with above; delete?)**

probes = names(datExpr)

inModule = is.finite(match(moduleColors, modules));

modProbes = probes[inModule];

modGenes = annot$NCBI_ID[match(modProbes, annot$Gene_ID)];

**#Select the corresponding Topological Overlap**

modTOM = TOM[inModule, inModule];

dimnames(modTOM) = list(modProbes, modProbes)

**#Export the network into edge and node list files Cytoscape can read. The paste**

cyt = exportNetworkToCytoscape(modTOM, edgeFile = paste("DSDRWW_T.3CytoscapeInput-edges-", paste(modules, collapse="-"), ".txt", sep=""), nodeFile = paste("DSDRWW_0.3CytoscapeInput-nodes-", paste(modules, collapse="-"), ".txt", sep=""), weighted = TRUE, threshold = 0.3, nodeNames = modProbes, altNodeNames = modGenes, nodeAttr = moduleColors[inModule]);

cyt = exportNetworkToCytoscape(modTOM, edgeFile = paste("DSDRWW_T.25CytoscapeInput-edges-", paste(modules, collapse="-"), ".txt", sep=""), nodeFile = paste("DSDRWW_0.25CytoscapeInput-nodes-", paste(modules, collapse="-"), ".txt", sep=""), weighted = TRUE, threshold = 0.25, nodeNames = modProbes, altNodeNames = modGenes, nodeAttr = moduleColors[inModule]);

cyt = exportNetworkToCytoscape(modTOM, edgeFile = paste("DSDRWW_T.2CytoscapeInput-edges-", paste(modules, collapse="-"), ".txt", sep=""), nodeFile = paste("DSDRWW_0.2CytoscapeInput-nodes-", paste(modules, collapse="-"), ".txt", sep=""), weighted = TRUE, threshold = 0.2, nodeNames = modProbes, altNodeNames = modGenes, nodeAttr = moduleColors[inModule]);

cyt = exportNetworkToCytoscape(modTOM, edgeFile = paste("DSDRWW_T.15CytoscapeInput-edges-", paste(modules, collapse="-"), ".txt", sep=""), nodeFile = paste("DSDRWW_0.15CytoscapeInput-nodes-", paste(modules, collapse="-"), ".txt", sep=""), weighted = TRUE, threshold = 0.15, nodeNames = modProbes, altNodeNames = modGenes, nodeAttr = moduleColors[inModule]);

cyt = exportNetworkToCytoscape(modTOM, edgeFile = paste("DSDRWW_T.1CytoscapeInput-edges-", paste(modules, collapse="-"), ".txt", sep=""), nodeFile = paste("DSDRWW_0.1CytoscapeInput-nodes-", paste(modules, collapse="-"), ".txt", sep=""), weighted = TRUE, threshold = 0.1, nodeNames = modProbes, altNodeNames = modGenes, nodeAttr = moduleColors[inModule]);

cyt = exportNetworkToCytoscape(modTOM, edgeFile = paste("DSDRWW_T.05CytoscapeInput-edges-", paste(modules, collapse="-"), ".txt", sep=""), nodeFile = paste("DSDRWW_0.05CytoscapeInput-nodes-", paste(modules, collapse="-"), ".txt", sep=""), weighted = TRUE, threshold = 0.05, nodeNames = modProbes, altNodeNames = modGenes, nodeAttr = moduleColors[inModule]);

cyt = exportNetworkToCytoscape(modTOM, edgeFile = paste("DSDRWW_T.01CytoscapeInput-edges-", paste(modules, collapse="-"), ".txt", sep=""), nodeFile = paste("DSDRWW_0.01CytoscapeInput-nodes-", paste(modules, collapse="-"), ".txt", sep=""), weighted = TRUE, threshold = 0.01, nodeNames = modProbes, altNodeNames = modGenes, nodeAttr = moduleColors[inModule]);

cyt = exportNetworkToCytoscape(modTOM, edgeFile = paste("DSDRWW_T.075CytoscapeInput-edges-", paste(modules, collapse="-"), ".txt", sep=""), nodeFile = paste("DSDRWW_0.075CytoscapeInput-nodes-", paste(modules, collapse="-"), ".txt", sep=""), weighted = TRUE, threshold = 0.075, nodeNames = modProbes, altNodeNames = modGenes, nodeAttr = moduleColors[inModule]);

cyt = exportNetworkToCytoscape(modTOM, edgeFile = paste("DSDRWW_T.070CytoscapeInput-edges-", paste(modules, collapse="-"), ".txt", sep=""), nodeFile = paste("DSDRWW_0.070CytoscapeInput-nodes-", paste(modules, collapse="-"), ".txt", sep=""), weighted = TRUE, threshold = 0.070, nodeNames = modProbes, altNodeNames = modGenes, nodeAttr = moduleColors[inModule]);

cyt = exportNetworkToCytoscape(modTOM, edgeFile = paste("DSDRWW_T.065CytoscapeInput-edges-", paste(modules, collapse="-"), ".txt", sep=""), nodeFile = paste("DSDRWW_0.065CytoscapeInput-nodes-", paste(modules, collapse="-"), ".txt", sep=""), weighted = TRUE, threshold = 0.065, nodeNames = modProbes, altNodeNames = modGenes, nodeAttr = moduleColors[inModule]);
