# Supplementary material for: Microarray analysis and scale-free gene networks identify candidate regulators in drought-stressed roots of loblolly pine (P. taeda L.)
Source: BMC Genomics. 2011 May 24;12:264. doi: 10.1186/1471-2164-12-264 (PMC3123330; doi:10.1186/1471-2164-12-264)
Supplement: Additional file 6 — Quantitative PCR analysis of highly differentially expressed genes and moderately expressed transcription factors. This file contains the results of qPCR analysis performed on 16 genes that were used to compare fold expression levels with those determined using PtGen2. RT-qPCR values were corrected to Actin 1 gene for each sample. For each treatment group three qPCR measurements were taken for each of four biological replicates and then averaged. Gene ID = the physical address of the spot on the array (metarow, metacolumn, row, column), UniScript = Fungen assembly contig ID. Microarray = log2 mean ratios and RT-qPCR = absolute fold difference after normalization to the P. taeda actin2 gene. [file 1471-2164-12-264-S6.PDF]

Additional File 6

RT-qPCR analysis of sixteen genes identified by PtGen2 as being either highly differentially expressed or as moderately expressed transcription factors.

Gene ID	Uniscript	Putative Function	Accession	Microarray	RT-qPCR
10.2.21.6	2_3931	chitinase	XP_002275386.1	12.1	2244
11.2.16.23	2_10347	metallothionein	AAT45001.1	11.8	157
1.3.16.12	2_5201	dehydrin	ACA51879.1	9.2	2969
4.1.22.8	2_8298	NBS/LRR	AAM28916.1	9.1	655
3.2.8.7	2_6459	DREB1	ABJ09421.1	2.3	3
7.2.16.23	2_8048	basic leucine zipper protein	XP_002263159.1	2	2
1.2.19.22	2_8167	ERF3	NP_175479.1	1.9	69
10.1.13.7	0_5245	P-type R2R3 Myb	AAB58314.1	1.8	393
11.3.19.9	2_8497	RING-H2 finger protein	EEF41575.1	1.6	17
5.2.15.14	2_3847	hydroxyproline-rich glycoprotein	NP_001064306.1	-8.1	-1.4
2.1.7.11	0_12961	terpinolene synthase	Q9M7D0	-6.1	-95
11.3.19.21	2_32	pinene synthase	AAO61228.1	-5.8	-98
7.3.19.20	2_2267	O-diphenol-O-methyl transferase	NP_190882.1	-5.7	-0.5
12.1.21.9	2_2508	class III HD-Zip	ABG73246.1	-2	-1
1.2.18.1	2_9495	WRKY	EEF31086.1	-1.5	-1.2
11.4.5.8	0_14551	ring finger protein	EEF51968.1	-1.6	-4.7
